# Exposure to Poverty and Productivity

**DOI:** 10.1371/journal.pone.0170231

**Published:** 2017-01-26

**Authors:** Patricio S. Dalton, Victor H. Gonzalez Jimenez, Charles N. Noussair

**Affiliations:** 1 Department of Economics, Tilburg University, Tilburg, The Netherlands; 2 Department of Economics, Eller College of Management, University of Arizona, Tucson, Arizona, United States of America; Middlesex University, UNITED KINGDOM

## Abstract

We study whether exposure to poverty can induce affective states that decrease productivity. In a controlled laboratory setting, we find that subjects randomly assigned to a treatment, in which they view a video featuring individuals that live in extreme poverty, exhibit lower subsequent productivity compared to subjects assigned to a control treatment. Questionnaire responses, as well as facial recognition software, provide quantitative measures of the affective state evoked by the two treatments. Subjects exposed to images of poverty experience a more negative affective state than those in the control treatment. Further analysis shows that individuals in a more positive emotional state exhibit less of a treatment effect. Also, those who exhibit greater attentiveness upon viewing the poverty video are less productive. The results are consistent with the notion that exposure to poverty can induce a psychological state in individuals that adversely affects productivity.

## Introduction

The state of poverty influences productivity in at least two different ways. On the one hand, financial constraints dampen physical and cognitive performance through nutritional deficiencies [1, 2], low educational quality [3, 4], and poor health conditions [5, 6], which in turn affect productivity. On the other hand, a recent literature underscoring the psychological aspects of poverty has identified additional channels through which poverty affects individual decisions in a way that can become counterproductive. These mechanisms include risk and time preferences [7] or individuals’ motivations and aspirations [8, 9]. According to [7], the economic and social conditions under which poor people live may lower their willingness to take risks and to forgo current income in favor of higher future incomes, even though the intrinsic time and risk preferences of the poor may be identical to those of wealthier people. One plausible explanation may be that the poor are more liquidity constrained. Because of this tighter constraint, if a poor individual has the choice between a current and a delayed payment in an experiment, he or she may opt for the current payment. Similarly, the anticipation of future liquidity constraints may also induce an individual to prefer a safe payment over a risky payment. Regarding aspirations, [8] observe that, due to lower access to credit, less influential contacts or less access to relevant information, poverty makes it harder for the poor to achieve a given outcome, ceteris paribus. This exacerbates the adverse effects of a behavioural bias that both poor and wealthier people may have in setting aspirations. As a consequence, the poor are more likely to choose a low aspiration level and effort relative to the best outcome they could achieve.

Our focus in this research is on a particular aspect of the psychology of poverty. There may be psychological effects arising from exposure to the poverty of others that an individual is in contact with, which are distinct from those arising directly from one’s own experience of poverty. We study whether the affective state associated with exposure to the poverty of others, on its own, leads to lower individual productivity. Such an effect would exist above and beyond the consequences of other difficulties that the poor face. We study the link between exposure to poverty of others and productivity in a controlled setting, where the effect can be isolated from other factors and affect can be precisely measured. We construct an experimental environment designed to induce the affective load associated with exposure to others’ poverty, but without the physical, social and economic consequences of one’s own poverty. We do so by providing individuals with minimal exposure to conditions of poverty suffered by others. We operate under the assumption that the emotions induced by longer, more intense, and more personal, exposure to poverty than those we create here would be at least as strong.

If exposure to the poverty of others reduces work performance, it would suggest that removing poor individuals from such exposure might increase their productivity. Indeed, in a recent paper, [10] show that young children assigned to the Moving to Opportunity program, in which poor American families are given vouchers which allow them to rent housing in more affluent areas, exhibited an increase in college attendance rates and income. These results corroborate some of the findings of [11]. The study finds that these improvements stem from a difference in the childhood experience of those assigned to the program. Among the aspects included in such experience, the authors highlight better education, greater safety, greater neighborhood satisfaction, and a lower incidence of single parent households. While one might presume that emotional factors are at work and contributing to the better outcomes of families in the program, the effect of improved emotional state cannot be distinguished from that of the other resources they have available.

We differ from the existing literature in that we study whether the affects associated with mere exposure to the poverty of others, rather than with the experience of one’s own poverty itself, have an effect on productivity. The exposure to poverty in our study is brief and not very intense. Nevertheless, we find that mere exposure to a video showing the reality of poverty for seven minutes has an effect on subsequent performance in a relatively simple task. It also induces a more negative emotional state. Detailed analysis of the data, however, suggests that the effect of exposure to poverty of others on performance is cognitive rather than emotional, as the exposure appears to impede the focus of attention on performing the task.

Our experiment has two treatments. In the *Poverty* treatment, subjects watch a video clip that illustrates the conditions faced by a family in a state of poverty. In the *Neutral* treatment, which serves as a control condition, subjects observe a neutral video, known from other studies to evoke no strong emotional response.

To measure individual productivity we use a real effort task. We employ the slider task introduced by [12], which consists of setting as many sliders as possible in the exact middle position of the available range, by moving a cursor with a computer mouse. The advantages of this task for our purposes are that it does not require specialized knowledge, the instructions are easy to follow, the output has no value to the experimenter (so that social preferences with regard to the experimenter are minimized as a consideration in participants’ effort decisions), and it has been widely used previously in experimental economics. This last feature facilitates comparison of any effect sizes that we observe to previous and future studies. An individual’s productivity is measured as the number of sliders that are correctly aligned in a 20-minute work period.

We register psychological affects in two ways. First, we administer the PANAS questionnaire to participants immediately after they view the video clip. This questionnaire provides a subjective self-evaluation of the current intensity of a number of specific emotions [13], and allows for the construction of broader indices describing more general affective states. Second, we use a facial recognition software package, called Noldus Facereader^*TM*^, to identify the intensity of the emotions evoked by the videos.

We find that the subjects randomly assigned to the *Poverty* treatment exhibit lower average performance than those assigned to the *Neutral* treatment. Moreover, images of poverty evoke higher self-reported scores on measures of negative affect and attentiveness. The physiological data from the facereading software confirms that exposure to poverty induces an emotional state of more negative valence than does the control. Further analysis shows that: i) The difference in productivity between the two treatments is greater for individuals in a less positive emotional state after watching the video, and ii) subjects who display greater attentiveness when viewing the images of poverty display lower productivity.

Our paper fits into a recent and active literature that focuses on the psychology of poverty. While previous research documents a relationship between affective state and poverty [7], we show that mere exposure to poverty of others can influence own affective state, which in turn affects productivity. Our work is also consistent with studies that associate positive emotional states with better performance in various tasks [14, 15, 16, 17, 18], in settings unrelated to poverty. It also relates to work that explores the role of poverty on cognition [19, 20, 21]. We find that subjects exposed to images of poverty report being more attentive after watching the video, and that those who were the most attentive displayed lower productivity. This suggests that for some individuals, exposure to poverty of others imposes a cognitive load that hampers their performance.

## Experimental design and procedures

Our experiment employs human subjects. Our protocol was not approved by an Institutional Review Board. However, we are fully in compliance with Dutch Law, which does not require social science research to receive prior approval from an IRB. Although Tilburg University does not have an institutionalized IRB, the Director of CentERLab or the Scientific Director of CentER screens and authorizes the content and purpose of all of the experiments taking place in the laboratory. This particular study was reviewed and approved by the Scientific Director of CentER, Professor Geert Duijsters, after the research was conducted. He formally confirmed that the study was conducted according to the principles expressed in the Declaration of Helsinki, and that this work complied with Dutch laws and Tilburg School of Economics and Management’s policy regarding the ethical treatment of human subjects. All subjects gave their signed written consent to participate in the study at the beginning of their experimental session, including consenting to be videotaped.

Our dataset consists of 15 experimental sessions conducted in June, 2014 at the CentERLab at Tilburg University in the Netherlands. All subjects were students at the university. We used z-Tree [22] to implement the experiment. Subjects were recruited via an online system. On average, a session lasted approximately 45 minutes. Between five and ten subjects took part in each session, and no subject participated more than once in the experiment.

There were two treatments, *Poverty* and *Neutral*, and upon arrival at the experimental laboratory, subjects were randomly assigned to one of the treatments. A total of 105 participants whose average age was 23, participated in the study, 55 in the *Poverty* treatment, and 50 in the *Neutral* treatment. In the *Poverty* treatment, subjects watched a video clip that depicted the struggles of a poor family living in a garbage dump in Moscow, Russia. In the *Neutral* treatment, subjects watched a video of the Alaskan landscape that is known not to evoke any emotion or mood, and has been used in psychological research to induce a state of neutrality [23]. We did not film the videos ourselves. They were publicly available online. The *Poverty* video is available in the following link https://www.youtube.com/watch?v=lDzhufj9GN0. A 2 minute version of the *Neutral* video is available in the following link https://www.youtube.com/watch?v=rbTCQrNOV_w. Both videos lasted for six to seven minutes. Subjects performed the experiment in individual soundproof cubicles. This allowed us to run both treatments within the same session while having each subject participate in only one treatment, thus avoiding confounds from session fixed effects [24].

After watching one of the videos, subjects had to complete a PANAS positive and negative affects schedule [13]. In this questionnaire, subjects stated the current subjective intensity, on a scale from one to five, of various affects. Ten negative and ten positive affects are included in this schedule. From the responses to these questions, we constructed scales of positive affect, negative affect, self-assurance, attentiveness, hostility, joviality, guilt, hostility, and fear. The questionnaire is reprinted in Supporting Information.

After completing this questionnaire, subjects performed a time consuming real effort task. We used the task introduced by [12], known as the slider task. It consists of setting the highest possible number of sliders, which are displayed on the subject’s computer screen, in the exact middle point of a pre-specified range, using their computer mouse to move a cursor. The task was unfamiliar to all participants and it entailed a cost of effort in terms of attention and patience.

Subjects assigned to either treatment faced the same piece-rate incentive in the slider task. The accurate completion of each slider increased an individual’s earnings by 5 Euro cents. Each session was divided into ten periods of 2 minutes each. All periods counted towards the subjects’ earnings.

Finally, subjects completed a questionnaire to gather information on demographic characteristics such as age, program of study, country of origin, previous exposure to poverty, gender, and indirect questions intended to measure family wealth and socioeconomic status. Previous exposure to poverty captures whether the subject traveled and/or lived in a poor country. We acknowledge that this measure is imperfect. However, we believe that is at least positively correlated with actual previous exposure to poverty. While it is certainly true that there is poverty in every European country, one can expect that ceteris-paribus, a person who has lived in or traveled to the developing world is more exposed to the type of images of poverty in the video, than someone who has not left the developed world. The questionnaire can be found in the supporting information. Identifying information was destroyed after the initial processing of the data and replaced with identifiers that did not permit the identity of a participant to be deduced.

Throughout the entire session, subjects were videotaped with their prior consent using the webcams on their computers. The videos were analysed later using the facial recognition software Noldus Facereader. The software locates 530 points on a subject’s face, and compares it to a database of several thousand annotated images. Facereader measures the conformity of the subject’s facial expression to each of the six universal emotions: Happiness, Anger, Sadness, Disgust, Scare and Surprise, as well as Neutrality. The facial expressions that correspond to the six basic emotions appear to be universal and innate, in that they are common across all cultures and across different primates [25, 26], as well as between blind and sighted humans [26, 27]. Facereader takes a reading every 1/30th of a second. The program also constructs a measure of valence, using the formula *Happiness* − *max*(*Anger*, *Sadness*, *Disgust*, *Scare*). In our analysis, we use the average reading of each emotion over the one minute interval before the video begins, as well as over the one minute interval after the video ends. The effect of the video on an individual is measured as the difference in the average in the earlier and later intervals.

## Results

### Performance

**Fig 1:**
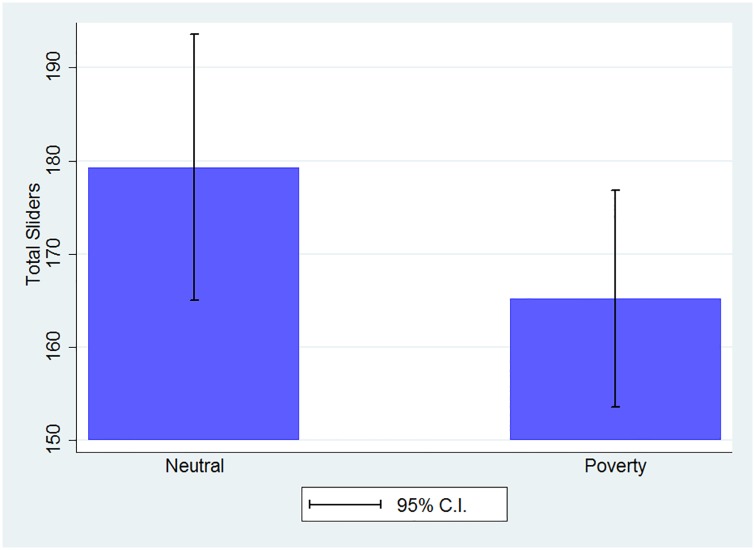
Average performance by treatment.

The measure of performance in our experiment is the number of sliders an individual correctly aligns over the course of a ten-period session. On average subjects solved 171.91 sliders with a standard deviation of 46.96 in a session. As illustrated in Fig 1, subjects in the *Poverty* treatment solved 165.2 sliders as compared to 179.3 sliders in the *Neutral* treatment. This difference is borderline significant (t(97,151) = 1.534, p = 0.063). A Kolmogorov-Smirnov test rejects the hypothesis that the number of sliders completed in the two treatments are drawn from the same distribution (KS-test, p<0.01). Performance in the Neutral treatment is significantly lower than that observed by [12], where subjects on average solved 222 sliders in 20 minutes. We attribute this difference to the fact that [12] employ tournament incentives that have been proven to increase effort [28].

Furthermore, the treatment effect on performance is significant once other sources of variation are controlled for. We estimate a regression of performance on condition dummies, covariates of wealth, and previous exposure to poverty. The estimates of this linear regression are presented in Table 1. The table shows that the effect of being assigned to *Poverty* is significant once the variables that capture the subject’s previous exposure to poverty and wealth are included. Ceteris paribus, a subject assigned to *Poverty* produces on average 1.54 less sliders in each two-minute round as compared to a subject assigned to the *Neutral* condition.

**Table 1:** Linear Regression of Performance on Treatment, Round and Control Variables.

	(1)	(2)	(3)
	Performance	Performance	Performance
Poverty	-1.410	-1.547 *	-1.544 *
	(-1.54)	(-1.68)	(-1.68)
Previous Exposure		0.724	0.731
		(1.38)	(1.38)
Wealth			-0.076
			(-0.12)
Round	0.703***	0.703***	0.703***
	(14.57)	(14.56)	(14.55)
Constant	14.06***	13.40***	13.45***
	(18.99)	(16.03)	(13.12)
*N*	1050	1050	1050
R^2^	0.133	0.142	0.142

Note: This table presents the estimates of an ordinary least squares regression of the statistical model *Performance*_*i*_ = *α*_0_ + *α*_1_*Poverty*_*i*_ + *α*_2_*PreviousExposure* + *α*_3_*Wealth* + *ϵ*_*i*1_. *Performance_*i*_* is the number of sliders solved per round. *Previous Exposure* is a variable that captures whether the subject traveled and/or lived in a poor country. *Wealth* is a variable that captures whether the subject’s parents have more than three cars and/or own more than two real estate properties. Clustered standard errors at the individual level.

*p<0.1;

** p<0.05;

***p<0.01.

**Fig 2:**
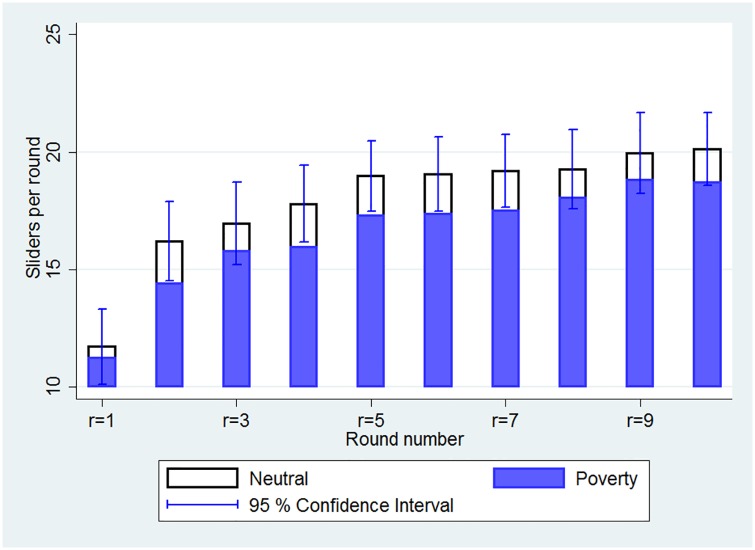
Average performance by round and treatment.

Fig 2 illustrates the performance gap between the treatments by round. This figure shows that the average number of sliders in every round is lower for subjects assigned to the *Poverty* treatment. Moreover, the figure suggests that the effect of the *Poverty* video on performance persists throughout the entire session. This may be either because the video itself affects performance throughout the session, or that the video has only a short-term effect in early rounds, but the performance in early rounds serves to anchor performance in later rounds.

### Affects

#### Self-reported measures

As described in the experimental design and procedures section, we administered the PANAS questionnaire [13] immediately after the subjects watched the video. Based on [29], we constructed, using the subjects responses, six emotional and affective scales, and two general dimension scales. Tables 2 and 3 present the mean and standard deviation of negative and positive affects in each treatment. The tables also report the average values of the affective scales and general dimension scales for both treatments.

**Table 2:** Average and Standard Deviation of Positive Affect Scales, by Treatment.

Affect	Poverty	Neutral	Z-Score
**Items**			
Interested	3.691	3.100	2.879***
	(1.143)	(1.119)	
Excited	2.145	2.600	-2.089**
	(1.070)	(1.021)	
Strong	2.927	2.620	1.388
	(1.190)	(1.019)	
Enthusiastic	2.273	2.780	-2.393 **
	(1.136)	(1.083)	
Proud	1.982	2.180	-1.304
	(1.259)	(1.091)	
Alert	3.291	2.680	2.258 **
	(1.247)	(1.393)	
Inspired	3.055	2.540	2.239 **
	(1.243)	(1.082)	
Determined	3.309	2.880	2.155**
	(1.094)	(1.014)	
Attentive	3.418	3.120	1.178
	(1.004)	(1.108)	
Active	2.709	3.080	-1.590
	(1.217)	(.9978)	
**Affective Scales**			
Joviality	4.41	5.38	-2.546 **
	(1.877)	(1.940)	
Self-Assurance	4.909	4.8	0.172
	(2.076)	(1.910)	
Attentiveness	13.709	11.78	3.006***
	(3.309)	(3.180)	
**General Dimension Scale**			
Positive Affects (PA)	28.80	27.58	2.236 **
	(7.631)	(7.655)	
N	55	50	

Note: This table presents the average score and standard deviation of each of the ten items representing positive affects in the PANAS questionnaire. The standard deviation of each item is presented in parentheses. The scale *Joviality* is constructed as the sum of the items *Excited* and *Enthusiastic*. The scale *Self-Assurance* is constructed as the sum of the items *Proud* and *Strong*. The scale *Attentiveness* is constructed as the sum of the items *Inspired*, *Determined* and *Attentive*. The row *Positive Affects* is the summation of the ten items. The column *Z-score* presents the statistic evaluating the rank sum difference between the two treatments. The significance is evaluated with the following significance levels.

*p<0.1;

** p<0.05;

*** p<0.01.

**Table 3:** Average and Standard Deviation of Negative Affect Scales, by Treatment.

Affect	Poverty	Neutral	Z-Score
**Items**			
Distressed	2.509	2.560	-0.126
	(1.008)	(1.204)	
Upset	2.782	1.620	4.789***
	(1.247)	(1.038)	
Guilty	2.218	1.420	3.749 ***
	(1.217)	(0.778)	
Scared	1.709	1.540	0.596
	(1.004)	(0.830)	
Hostile	1.818	1.400	2.200 **
	(.9934)	(0.722)	
Irritable	2.036	2.040	0.027
	(1.010)	(1.039)	
Ashamed	2.182	1.440	3.401***
	(1.178)	(0.753)	
Nervous	1.982	2.100	-0.622
	(1.088)	(1.064)	
Jittery	2.145	2.000	0.822
	(0.924)	(0.939)	
Afraid	1.655	1.560	0.458
	(0.920)	(0.829)	
**Affective Scales**			
Fear	5.509	5.1	1.028
	(2.167)	(2.121)	
Guilt	4.4	2.86	3.631***
	(2.231)	(1.343)	
Hostility	1.818	1.400	2.200 **
	(.993)	(0.722)	
**General Dimension Scale**			
Negative Affects (NA)	21.036	17.680	2.728 ***
	(6.327)	(5.859)	
N	55	50	

Note: This table presents the average score and standard deviation of each of each of the ten items representing negative affects in the PANAS questionnaire.The standard deviation of each item presented in parentheses. The scale *Fear* is constructed as the sum of the items *Afraid*, *Scared* and *Jittery*. The scale *Guilt* is constructed as the sum of the items *Guilt* and *Ashamed*. The scale *Hostile* is represented by the item *Hostility*. The row *Negative Affects* is the summation of the ten items. The column *Z-Score* presents the statistic evaluating the rank sum difference between the two treatments. The significance is evaluated with the following significance levels.

*p<0.1;

** p<0.05;

***p<0.01.

These tables show that the *Poverty* treatment yields a higher score on the general dimension scale of negative affects (referred to as NA from here onward), compared to the *Neutral* treatment (p<0.01). This difference stems from the higher score of the Guilt scale, composed by the items *Guilty* and *Ashamed* (p<0.001) and the higher scores of the items *Hostile* (p<0.05), and *Upset* (p<0.001), under *Poverty*.

Moreover, the *Poverty* treatment also yields a higher score on the general dimension scale of positive affects (PA from here onward), than under *Neutral* (p = 0.025). This difference between treatments is driven by a higher score on the attentiveness scale, composed of the items *Alert*, *Determined* and *Interested*, in *Poverty* (p<0.05). Note that even though the Joviality scale, composed of *Enthusiastic* and *Excited*, exhibits a lower score under *Poverty* (p = 0.011), the direction of the difference in PA between treatments shows that this difference is not as large as that displayed by attentiveness.

*Poverty* induces higher average negative affects compared to the PANAS scores obtained under typical natural conditions. The average score of 21.036 in NA after watching the *Poverty* video is significantly larger than a typical baseline average of NA 14.8 reported by [13]. However, the *Poverty* treatment does not induce higher average positive affect than 29.7, the score observed by [13].

Taken together, these results show that the *Poverty* video induces a considerable increase on the score of both positive and negative affects as compared to the *Neutral* video. On the one hand the *Poverty* video evokes higher scores on guilt and hostility. On the other hand, the video increases the score related to attentiveness.

#### Physiological Measure of Emotional State

**Fig 3:**
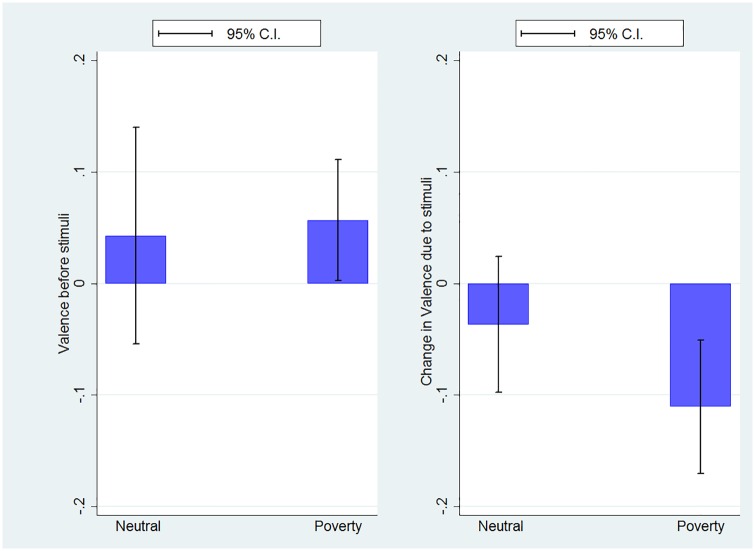
Initial value and change in valence from before to after the video was shown.

The Facereader data indicate that before the videos are played, there are no significant differences in the physiological measures of emotions between treatments. We report no significant difference across treatments in neutrality (p = .9873), sadness (p = 0.1591) happiness (p = .757), anger (p = .7411), scare (p = .4343) or disgust (p = .6738). As a consequence and as can be seen in Fig 3, there is no significant difference in the emotional valence before presentation of the videos (p = 0.782).

The descriptive statistics of the subject’s physiological reaction due to the videos, measured as the difference between the one-minute interval before the beginning of the video and the one-minute interval immediately after it has finished, are presented in Table 4. These statistics, along with Fig 3, show that the *Poverty* video induces a more negative valence (one sided t-test, p = .055). This finding is in agreement with the self-reported data, in that the *Poverty* treatment induces a more negative emotional state than the *Neutral* treatment. This difference is driven by the specific emotions of *Scare* (one sided t-test, p = .039) and *Sadness* (one sided t-test, p = .057). These patterns, coupled with the PANAS results, reveal that the *Poverty* video increases a number of negative emotions such as fear, guilt, sadness, shame and upset. Whether these differences in affects correlate with lower performance is addressed in the next section.

**Table 4:** Changes in Physiological Measure of Emotions from Before to After the Video, both Treatments.

Emotion	Poverty	Neutral	Difference
Neutral	-.0011	0.048	-.058
	(.213)	(0.294)	(.066)
Happy	-.068	-0.051	.-017
	(.125)	(0.122)	(.033)
Sad	0.037	-0.002	.039*
	(0.098)	(0.077)	(.024)
Angry	0.002	-0.002	.004
	(0.091)	(0.062)	(.025)
Surprised	0.007	0.027	-.020
	(0.097)	(0.090)	(.022)
Scared	0.010	-0.006	.015**
	(0.036)	(0.019)	(.008)
Disgusted	-0.003	-0.004	.001
	(0.016)	(0.010)	(.003)
Valence	-0.111	-0.037	-.073*
	(0.185)	(0.134)	(.045)
N	39	21	21

Note: This table presents the difference in average intensity, between before and after the stimulus was presented to the subjects for the 7 emotions analyzed by the facereader software. The standard deviation is given in Parentheses. The row *Valence* contains the change in average valence from before to after the stimulus was presented to the subjects. Valence is calculated as *Happiness* − *max*(*Sadness*, *Anger*, *Scare*, *Disgust*). The column labeled *Difference* gives the average difference in emotional intensity between the *Poverty* and *Neutral* treatments. The significance of *Difference* is evaluated with a one sided t-test.

*p<0.1;

** p<0.05;

***p<0.01.

## What Moderates the Effect of Exposure to Poverty of Others?

The results reported in the previous section indicate that subjects assigned to *Poverty* experience lower average performance. Moreover, participants in the two treatments exhibit differences in affective scales: under *Poverty* they have a more negative, self-reported and physiologically measured, affective state. They also have higher scores on the attentiveness scale, and lower scores on the joviality scale. To investigate whether the treatment difference in performance varies depending on an individual’s affective state, we employ a moderation analysis [30]. A variable is said to moderate a treatment effect if higher values of the variable are correlated with a weaker treatment effect. For example, suppose that the sample of all participants is divided into two subsamples. In one subsample are those with a relatively positive prior emotional state, and in the other those in a relatively negative one. If positive emotional valence moderates the treatment effect, the difference between treatments would be greater for the subsample in the relatively negative state than that in the more positive state.

Table 5 presents the estimates of the statistical model,

$$Performance_{i}=\alpha_{0}+\alpha_{1}Poverty+\alpha_{2}A_{i}+\alpha_{3}A_{i}*Poverty+\Gamma^{\prime }X+\epsilon_{i},$$

where *Poverty* is a dummy variable that equals 1 under the Poverty treatment and *A*_*i*_ is a variable that captures an affective measure for subject *i*. Throughout the analysis, *A*_*i*_ may represent a single affect or emotion, an affective scale as in [29], physiological valence, or a general dimension scale. We begin the moderation analysis using the responses of the PANAS. Specifically, we estimate the statistical model with the dimension scales NA or PA representing *A*_*i*_. These composite scores capture the total variation of self-reported affects. The coefficients on the variable *Poverty* * *A*_*i*_ reveal whether the scale or emotion in question is a moderating factor. The first two columns of Table 5 show that these scales do not moderate the effect of treatment on performance. Hence, the lower performance of those assigned to *Poverty* does not depend on the variation in the general score of positive or negative affects between treatments.

**Table 5:** Moderation Analysis, Self-Reported Affects.

	(1)	(2)	(3)	(4)	(5)	(6)	(7)	(8)
	Performance	Performance	Performance	Performance	Performance	Performance	Performance	Performance
Poverty	0.530	-5.739*	0.465	0.046	-0.419	-7.584***	-3.045	-4.543
	(2.905)	(2.959)	(2.382)	(1.932)	(2.007)	(2.297)	(2.098)	(3.356)
A_*i*_	0.0678	-0.174*	0.141	0.929	0.235	-0.654**	-0.520**	-0.389*
	(0.097)	(0.078)	(0.245)	(0.880)	(0.445)	(0.308)	(0.254)	(0.210)
Poverty* A_*i*_	-0.110	0.154	-0.377	-1.089	-0.339	1.225**	0.321	0.275
	(0.130)	(0.101)	(0.371)	(1.056)	(0.503)	(0.436)	(0.359)	(0.271)
Previous Exposure	0.767	0.653	0.764	0.738	0.759	0.801	0.657	0.628
	(0.533)	(0.555)	(0.535)	(0.539)	(0.525)	(0.540)	(0.538)	(0.540)
Wealth	-0.0716	-0.198	-0.0478	-0.0768	-0.103	-0.327	-0.179	-0.111
	(0.613)	(0.636)	(0.612)	(0.613)	(0.618)	(0.635)	(0.634)	(0.620)
Round	0.703***	0.703***	0.703***	0.703***	0.703***	0.703***	0.703***	0.703***
	(0.048)	(0.048)	(0.048)	(0.048)	(0.048)	(0.048)	(0.048)	(0.048)
Constant	12.21***	18.42***	12.68***	12.14***	12.77***	17.10***	16.09***	18.15***
	(2.218)	(2.574)	(1.822)	(1.438)	(1.788)	(2.130)	(1.676)	(2.762)
A_*i*_	NA	PA	Fear	Hostility	Guilt	Joviality	Self-Assesement	Attentiveness
N	1050	1050	1050	1050	1050	1050	1050	1050
adj. (R^2^)	0.143	0.165	0.145	0.147	0.142	0.179	0.156	0.164

Note: This table presents the estimates of an ordinary least squares regression of the statistical model *Performance*_*i*_ = *α*_0_ + *α*_1_ * *Poverty* + *α*_2_ * *A*_*i*_ + *α*_3_*A*_*i*_ * *Poverty* + Γ′*X*_*i*_ + *ϵ*_*i*1_. *Effort_*i*_* is the number of sliders solved per round. *T*_*i*_ is the assignment to the *Neutral* or the *Poverty* condition, *A*_*i*_ is a composite index of self-reported affects. The matrix *X*_*i*_ contains the variables *Previous Exposure*, which captures whether the subject traveled or/and lived in a poor country and *Wealth*, which is a variable that indicates whether the subject’s parents had more than 3 cars or/and more than 2 real estate properties. Standard errors are clustered at the level of the individual subject.

*p<0.1;

** p<0.05;

***p<0.01.

The moderation analysis also shows that the Joviality scale moderates the effect of the *Poverty* treatment on performance. This can be seen in column 6 of the table. Specifically, subjects reporting higher scores on the items *enthusiastic* and *excited* after watching the videos exhibit a smaller difference in performance between treatments. Finally, we report that the self-reported responses representing Fear, Hostility, Guilt, Self-Assessment and Attentiveness do not moderate the lower performance due to the video, even when some of these scales display a significant difference between treatments.

Additionally, we investigate the role of the physiological measures of emotions as moderators. We perform the moderation analysis using two different representations of emotional state using the facereader data. The first one is the absolute emotional state before the video is played, and the second one is the change in emotional state from before to after the video is played.

Table 6 reports whether the emotional state before viewing the video affects performance. The findings suggest that negative valence before the videos are presented moderate the treatment effect. In other words, subjects in a more negative prior emotional state experience a smaller reduction in performance from the *Poverty*, relative to the *Neutral*, condition. In particular, higher measures of happiness and higher values of sadness have a moderating effect.

**Table 6:** Moderation Analysis, Physiological Measures Taken Before Video is Played.

	(1)	(2)	(3)	(4)	(5)	(6)	(7)
	Performance	Performance	Performance	Performance	Performance	Performance	Performance
Poverty	-0.642	1.687	-4.550 *	-1.450	-2.182	-1.834	-0.948
	(1.313)	(1.421)	(2.290)	(1.735)	(1.741)	(1.654)	(1.442)
A_*i*_	12.16	16.29 *	-43.55**	5.558	-12.71	-36.65	-55.64
	(8.352)	(8.192)	(19.82)	(12.646)	(20.686)	(91.78)	(51.489)
Poverty*A_*i*_	-18.28**	-22.59***	46.71**	-0.0519	14.31	48.81	-205.2***
	(8.486)	(8.340)	(21.81)	(13.567)	(21.508)	(97.480)	(71.652)
Previous Exposure	0.644	0.486	0.315	0.553	0.525	0.621	0.705
	(0.634)	(0.624)	(0.775)	(0.715)	(0.699)	(0.720)	(0.641)
Wealth	-1.219	-1.023	-1.675*	-1.139	-1.180	-1.319	-1.622 *
	(0.867)	(0.809)	(0.896)	(0.909)	(0.976)	(0.925)	(0.845)
Round	0.712***	0.712***	0.712***	0.712***	0.712***	0.712***	0.712***
	(0.072)	(0.072)	(0.072)	(0.072)	(0.072)	(0.072)	(0.072)
Constant	13.99***	12.11***	18.14***	14.24***	15.12***	14.89***	15.17***
	(1.686)	(1.492)	(2.833)	(2.134)	(2.293)	(2.082)	(1.904)
A_*i*_	Valence	Happy	Sad	Angry	Surprised	Disgusted	Scared
*N*	610	610	610	610	610	610	610
adj. *R*^2^	0.239	0.249	0.205	0.160	0.158	0.157	0.227

Note: This table presents the estimates of an ordinary least squares regression of the statistical model *Performance*_*i*_ = *α*_0_ + *α*_1_ * *Poverty* + *α*_2_ * *A*_*i*_ + *α*_3_*A*_*i*_ * *Poverty* + Γ′*X*_*i*_ + *ϵ*_*i*1_. *Effort_*i*_* is the number of sliders solved per round. *T*_*i*_ is the assignment to the *Neutral* or the *Poverty* condition, *A*_*i*_ is a composite index of self-reported affects. The matrix *X*_*i*_ contains the variables *Previous Exposure*, which captures whether the subject traveled or/and lived in a poor country and *Wealth*, which is a variable that indicates whether the subject’s parents had more than 3 cars or/and more than 2 real estate properties. Standard errors are clustered at the level of the individual subject.

*p<0.1;

** p<0.05;

***p<0.01.

Furthermore, Table 7 presents the moderation analysis for the change in emotional state due to the videos. The results show that a more positive (or less negative) change in valence from before to after watching the video moderates the treatment effect. Hence, individuals experiencing a less negative change in valence after watching the video, exhibit a smaller difference in performance between treatments. This effect is led by the emotion of fear. For those experiencing relatively high levels of fear, there is a larger difference in performance after viewing the *Poverty* than the *Neutral* video.

**Table 7:** Moderation Analysis, Change in Physiological Measures Due to Video.

	(1)	(2)	(3)	(4)	(5)	(6)	(7)
	Performance	Performance	Performance	Performance	Performance	Performance	Performance
Poverty	0.0270	0.206	-0.220	-1.824	-2.891	-2.126	-0.675
	(1.202)	(1.386)	(2.155)	(1.601)	(1.836)	(1.624)	(1.488)
A_*i*_	-21.27*	16.96***	10.12	5.256	-10.32	-207.2	2171.3***
	(11.427)	(5.011)	(23.710)	(9.906)	(10.798)	(288.924)	(646.210)
Poverty* A_*i*_	26.60**	-18.32**	-19.90	2.871	15.47	119.4	-2176.0***
	(11.076)	(8.245)	(24.392)	(11.884)	(11.227)	(323.114)	(645.029)
Previous Exposure	0.135	0.874	0.453	0.591	0.432	0.674	0.713
	(0.613)	(0.675)	(0.653)	(0.688)	(0.725)	(0.640)	(0.665)
Wealth	-1.029	-1.490 *	-1.338	-1.377	-1.134	-1.597 *	-1.747**
	(0.826)	(0.863)	(0.837)	(0.913)	(0.865)	(0.872)	(0.835)
Round	0.703***	0.705***	0.705***	0.705***	0.705***	0.705***	0.705***
	(0.072)	(0.070)	(0.070)	(0.070)	(0.070)	(0.070)	(0.070)
Constant	14.00***	13.07***	14.50***	14.82***	15.66***	15.85***	14.33***
	(1.542)	(1.639)	(2.513)	(1.935)	(2.198)	(2.078)	(1.879)
A_*i*_	Valence	Happy	Sad	Angry	Surprised	Disgusted	Scared
*N*	600	620	620	620	620	620	620
adj. *R*^2^	0.247	0.221	0.173	0.167	0.170	0.174	0.186

Note: This table presents the estimates of an ordinary least squares regression of the statistical model *Performance*_*i*_ = *α*_0_ + *α*_1_ * *Poverty* + *α*_2_ * *A*_*i*_ + *α*_3_*A*_*i*_ * *Poverty* + Γ′*X*_*i*_ + *ϵ*_*i*1_. *Effort_*i*_* is the number of sliders solved per round. *T*_*i*_ is the assignment to the *Neutral* or the *Poverty* condition, *A*_*i*_ is a composite index of self-reported affects. The matrix *X*_*i*_ contains the variables *Previous Exposure*, which captures whether the subject traveled or/and lived in a poor country and *Wealth*, which is a variable that indicates whether the subject’s parents had more than 3 cars or/and more than 2 real estate properties. Standard errors are clustered at the level of the individual subject.

*p<0.1;

** p<0.05;

***p<0.01.

These two results illustrate that subjects experiencing lower emotional valence due to the *Poverty* video also exhibit lower performance levels, compared to subjects assigned to *Neutral*. Moreover, these declines in performance worsen with the degree of positive valence that a subject assigned to *Poverty* exhibits before the video is displayed.

## Do Affects and Emotions Mediate the Decrease in Productivity?

In this section we consider whether the effect of the videos on emotional state accounts for some or all of the difference in productivity between treatments. We employ the mediation analysis developed by [31] to evaluate the extent to which affective states induced by the videos mediate the lower performance. To that end, we estimate the following system of equations:

$$A_{i}=\beta_{0}+\beta_{1}Poverty+C^{\prime }X_{i}+\epsilon_{i1},$$

$$Performance_{i}=\alpha_{0}+\alpha_{1}Poverty+\alpha_{2}A_{i}+D^{\prime }X_{i}+\epsilon_{i2}.$$

The estimates of this system of equations intend to isolate two effects. The first is given by *α*_1_, which measures the direct effect of treatment on performance $\hat{\alpha_{1}}$. In other words, this is the effect on performance of being assigned to a treatment once the relationship between variations in affect and performance is accounted for. The second effect is the indirect effect of treatment on performance via changes in affect $\hat{\delta_{i}}=\hat{\alpha_{2}}\hat{\beta_{1}}$. The assumptions required for this interpretation of the estimates [31], are

$$\left\{Performance_{i}^{\prime },A_{i}\right\}⊥Poverty|X_{i},$$

and

$$Performance_{i}^{\prime }⊥A_{i}|X_{i},Poverty.$$

Where $Performance_{i}^{\prime }\neq Performance$. The first equation of the assumption states that the treatment assignment is statistically independent of potential performance outcomes and mediators given pre-treatment confounders. In our experiment this assumption holds due to the treatment randomization. The second equation states that the observed mediator is statistically independent given treatment assignment and pre-treatment confounders. This means that the mediator is regarded as if it were randomized over treatments. Evidence that this assumption holds in our sample is provided using the Facereading data before the video is displayed. As stated in Section 3, we find no differences between treatments in Physiological measures before the video was displayed. The total effect of treatment is the sum of the direct and indirect effects. We use the Quasi-Bayesian Monte Carlo approximation of [32] to make statistical inference about $\hat{\delta_{i}}$. The parameters reported are the average of 100 draws.

Tables 8 and 9 present the estimates, using the self-reported scales to represent *A*_*i*_. The findings suggest that neither of the general dimension scales, NA or PA, mediates the effect of the treatment on performance. Moreover, Table 9 shows that among the affective scales that exhibit significant differences between treatments, only the attentiveness scale is a significant mediator of the treatment effect. Specifically, this scale mediates 31% of the total treatment effect.

**Table 8:** Results of the Mediation Analysis with the General Dimension Scales PA and NA.

	(1)	(2)	(3)
	Performance	Performance	Performance
Mediator: *A*_*i*_ = NA			
$\hat{\delta_{i}}$ (Mediation Effect)	.00048	-.0372	-.0046
$\hat{\alpha_{1}}$ (Direct Effect)	-1.338	-1.285	-1.653 *
Total Effect	-1.338	-1.322	-1.657 *
Mediator: *A*_*i*_ = PA			
*δ*_*i*_ (Mediation Effect)	-.112	-.135	-.170
$\hat{\alpha_{1}}$ (Direct Effect)	-1.224	-1.174	-1.482
Total Effect	-1.337	-1.310	-1.652 *
Wealth	NO	YES	YES
Previous Exposure	NO	NO	YES
Observations	1050	1050	1050

Note: This table presents the average of 100 draws of a Monte Carlo Simulation using the sampling distribution of $\hat{\alpha_{2}}\hat{\beta_{1}}$ and $\hat{\alpha_{1}}$ which are estimated through multiple Least Squares of the system equatons composed by *A*_*i*_ = *β*_0_ + *β*_1_*Poverty*_*i*_ + *C*′*X*_*i*_ + *ϵ*_*i*1_ and $Performance_{i}=\alpha_{0}+\alpha_{1}Poverty_{i}+\alpha_{2}^{\prime }A_{i}+D^{\prime }X_{i}+\epsilon_{i2}$. *Performance_*i*_* is the number of sliders solved per round. *A*_*i*_ is the total score of the Negative Affects elicited through the PANAS questionnaire in the top panel of the table and the total score of the Positive Affects elicited through the PANAS questionnaire in the top panel of the table. *Previous Exposure* is a variable that captures whether the subject traveled and/or lived in a poor country. *Wealth* is a variable that captures whether the subject’s parents had more than 3 cars or/and more than 2 real estate properties. Clustered standard errors at the individual level.

*p<0.1;

** p<0.05;

***p<0.01.

**Table 9:** Mediation Analysis for Self-Reported Affect Scales.

	(1)	(2)	(3)
	Performance	Performance	Performance
Mediator: *A*_*i*_ = Joviality			
$\hat{\delta_{i}}$ (Mediation Effect)	.069	.0591	.033
$\hat{\gamma_{i}}$ (Direct Effect)	-1.401	-1.483	-1.561 **
Total Effect	-1.331	-1.424	-1.528 *
Mediator: *A*_*i*_ = Fear			
$\hat{\delta_{i}}$ (Mediation Effect)	-.0171	-.031	-.035
$\hat{\gamma_{i}}$ (Direct Effect)	-1.316	-1.395	-1.504 *
Total Effect	-1.333	-1.427	-1.540 *
Mediator: *A*_*i*_ = *Guilt*			
$\hat{\delta_{i}}$ (Mediation Effect)	-.0145	-.022	-.052
$\hat{\gamma_{i}}$ (Direct Effect)	-1.327	-1.405	-1.500
Total Effect	-1.342	-1.427	-1.552 *
Mediator: *A*_*i*_ = Attentiveness			
$\hat{\delta_{i}}$ (Mediation Effect)	-.501 **	-.549***	-.559 ***
$\hat{\gamma_{i}}$ (Direct Effect)	-.838	-.788	-1.126
Total Effect	-1.339	-1.338*	-1.685 *
Wealth	NO	YES	YES
Previous Exposure	NO	NO	YES
Observations	1050	1050	1050

Note: This table presents the average of 100 draws of a Monte Carlo Simulation using the sampling distribution of $\hat{\alpha_{2}}\hat{\beta_{1}}$ and $\hat{\alpha_{1}}$ which are estimated through multiple Least Squares of the system of models composed by *A*_*i*_ = *β*_0_ + *β*_1_*Poverty*_*i*_ + *CX*_*i*_ + *ϵ*_*i*1_ and $Performance_{i}=\alpha_{0}+\alpha_{1}T_{i}+\alpha_{2}^{\prime }A_{i}+DX_{i}^{\prime }+\epsilon_{i2}$. *Performance_*i*_* is the number of sliders per round. *A*_*i*_ is the guilt scale. *A*_*i*_ is the *Joviality* scale at the top panel, *Fear* at the top-middle panel, *Guilt* at the bottom-middle panel and Attentiveness at the bottom panel. *Previous Exposure* is a variable that captures whether the subject traveled and/or lived in a poor country. *Wealth* is a variable that captures whether the subject’s parents had more than 3 cars or/and more than 2 real estate properties. Clustered standard errors at the individual level.

*p<0.1;

** p<0.05;

***p<0.01.

We also study the role of the physiological measures as mediators. We report the estimates of happiness and valence as mediators in Table 10. We find that emotional valence does not mediate the effect of the *Poverty* video on performance. Nevertheless, we find that the specific emotion of happiness mediates the effect of the treatment, after controlling for some measures of previous exposure to poverty. Happiness mediates nearly 27% of the total effect in one specification, but this mediating effect disappears once we control for the degree of prior exposure to poverty, indicating that the effect is not robust. Finally, we find no evidence that the emotions *sadness*, *scare*, *disgust*, *surprise* or *anger* mediate the treatment difference.

**Table 10:** Mediation Analysis Table for the Physiological Variables of Happiness and Emotional Valence.

	(1)	(2)	(3)
	Performance	Performance	Performance
Mediator: *A*_*i*_ = Happiness			
$\hat{\delta_{i}}$ (Mediation Effect)	-.519	-.655*	-.564
$\hat{\gamma_{i}}$ (Direct Effect)	-.985	-.979	-1.149
Total Effect	-1.504	-1.635	-1.714
Mediator: *A*_*i*_ = Valence			
$\hat{\delta_{i}}$ (Mediation Effect)	-.395	-.422	-.369
$\hat{\gamma_{i}}$ (Direct Effect)	-1.093	-.948	-1.336
Total Effect	-1.487	-1.370	-1.705
Wealth	NO	YES	YES
Previous Exposure	NO	NO	YES
Observations	620	620	620

Note: This table presents the average of 100 draws of a Monte Carlo Simulation using the sampling distribution of $\hat{\alpha_{2}}\hat{\beta_{1}}$ and $\hat{\alpha_{1}}$ which are estimated through multiple Least Squares of the system of models composed by *A*_*i*_ = *β*_0_ + *β*_1_*Poverty*_*i*_ + *CX*_*i*_ + *ϵ*_*i*1_ and $Performance_{i}=\alpha_{0}+\alpha_{1}T_{i}+\alpha_{2}^{\prime }A_{i}+DX_{i}^{\prime }+\epsilon_{i2}$. *Performance_*i*_* is the number of sliders per round. *A*_*i*_ is the guilt scale. *A*_*i*_ is the *Happiness* measured by facereader at the top panel and *Valence* as measured by facereader at the bottom panel. *Previous Exposure* is a variable that captures whether the subject traveled and/or lived in a poor country. *Wealth* is a variable that captures whether the subject’s parents had more than 3 cars and/or more than 2 real estate properties. Clustered standard errors at the individual level.

*p<0.1;

** p<0.05;

***p<0.01.

## Conclusion

In this paper, we have presented evidence consistent with the notion that the affective state associated with exposure to the poverty of others can decrease individual productivity. We required participants in our study to view a video and then had them perform a task that required effort and concentration. In the *Poverty* treatment, the video exposed participants to images of poverty, and in the control treatment, a neutral video was shown instead. Subjects assigned to the *Poverty* treatment exhibited lower average productivity compared to subjects that were in the *Neutral* condition.

The *Poverty* video induces a more negative emotional state on the part of viewers, as measured both by self-reports and facial recognition software. The *Poverty* video increases the self-reported levels of some emotions that form components of a positive scale, attentiveness, but we do not view this as a positive emotional state, as for example we would view joy or satisfaction. Poverty does not increase the physiological measures of positive emotions registered with Facereader. The affective states measured on the PANAS scales of attentiveness, guilt, and hostility are greater under the *Poverty* than under the *Neutral* treatment. The facial recognition software reports that the *Poverty* video evokes greater fear and sadness than the *Neutral* video. These patterns show that (i) exposure to poverty of others, (ii) own emotional state, and (iii) own productivity, are related.

A moderation analysis allows us to consider who is more susceptible to the treatment effect. It reports that those who score higher on the Joviality scale after viewing a video exhibit a smaller difference between the two treatments. Similarly, the physiological data indicate that those in a more positive emotional state after watching the video are less susceptible to the treatment effect. This pattern suggests that an overall positive emotional state is a buffer against the adverse consequences of exposure to poverty of others.

A mediation analysis allows us to investigate the causal nature of these relationships. We conclude that the treatment effect of the *Poverty* video is mediated by self-reported measures of attentiveness. Specifically, those paying relatively more attention to the *Poverty* video, experience lower subsequent productivity. However, those who seem to avert their attention when being exposed to poverty experience a less detrimental impact from the exposure. This finding is in line with those of [21] and [20], in which individuals in the state of poverty exhibit an impediment in their cognitive function, something that the authors describe as “tunneling”. In our framework we provide evidence that exposure to the poverty of others diverts individuals’ attention, and this in turn decreases their performance in a subsequent productive task.

We view our study as a proof of principle, illustrating that mere exposure to the poverty of others can lead to a decrease in productivity. We have shown that very brief low-intensity exposure can have an effect on a task performed immediately after the exposure. We do not know for sure what would be the effect on productivity if the length and intensity of exposure were both increased to the scales that exist in developing countries or in underprivileged neighborhoods outside the laboratory. However, one can well imagine that the effect could be stronger and more long-lasting, unless individuals become less sensitive to its effect as they receive more exposure. Whether the effect of mere exposure to poverty of others would be strong and durable enough to have a long-term effect on work performance remains to be established in future research.

It would be interesting to study the effect of other videos that evoke strong emotions on productivity. They may have a similar effect on output as the video we chose because they induce similar emotions. However, the fact that we also observe a direct effect of our *Poverty* video on productivity means that emotions are not the only channel whereby the video is exerting its effect. There is a specific effect of the content of the video, once emotions are controlled for. It is certainly possible, and perhaps likely, that videos with different content would also induce a direct effect on productivity. It is certainly not the case that exposure to poverty is the only type of exposure that would have an effect of output. We make no claims that exposure to poverty is the only potential cause of low productivity, there are certainly many influences on productivity, and some of these presumably have emotional substrates as well.

The task that subjects have to perform in our experiment is one that requires attention and focus, but it is not cognitively demanding and has a repetitive aspect. Future studies could determine whether the same results hold for tasks that require a stronger application of cognitive abilities. In addition, the subject pool in this experiment was composed by university students who may have a higher or lower proclivity to be affected by images of poverty than other populations. Further studies could explore, by varying the characteristics of the sample of subjects, which groups might be more or less affected.

## Supporting Information

S1 Appendix Experimental instructions. PANAS Questionnaire. Socio-Economic Status Questionnaire.
(DOCX)

S1 Replication Code Stata codes used for data analysis. (DO)

S1 Dataset Dataset used for the analysis in Stata. (DTA)
