# Supplementary material for: Exposure to Poverty and Productivity
Source: PLoS One. 2017 Jan 26;12(1):e0170231. doi: 10.1371/journal.pone.0170231 (PMC5268424; doi:10.1371/journal.pone.0170231)
Supplement: S1 Appendix — PANAS Questionnaire. Socio-Economic Status Questionnaire. (DOCX) [file pone.0170231.s001.docx]

**Experimental instructions**

This is an experiment in the economics of decision-making. The instructions are simple and if you follow them carefully you might earn a considerable amount of money, which will be paid to you via Bank transfer at the end of the experiment. The amount of payment you receive depends on your decisions. The currency used in the experiment is Euros.

Once the experiment has started, no one is allowed to talk to anybody other than the experimenter. Anyone who violates this rule will lose his or her right to participate in this experiment. If you have further questions when reading these instructions please do not hesitate to raise your hand and formulate the question to the experimenter.

What do you have to do?

First we would like you to watch a video clip. The video will be displayed in your computer screen. Please make yourself comfortable: the clip will last about 10 minutes and I will have more instructions for you afterwards. Don't forget to use the provided headphones.

[6 - 7 minutes: Video (6 minutes for Neutral video, 7 minutes 45 seconds for Poverty video]

Thanks for watching, please fill out questionnaire 1 in and answer it to the best of your ability. When you are all done we will move to the next stage.

For the next task we will ask you to move your keyboard far from your reach. Now, you will have ten rounds of two minutes each to position 48 sliders at the exact middle using only the mouse. This is, you need to use the mouse to move the indicator of the slider until the digit located at the right is 50. You will be paid 10 cents per correctly positioned slider. Using the keyboard to complete the task is forbidden, anyone using the keyboard will be asked to leave the room. An example is provided

Please fill in the last questionnaire, answer as truthfully as you can and feel free to raise your hand if anything is unclear. Please note that as with the rest of your input today, your questionnaire answers are entirely anonymous: we will only link your answers to the specific computer ID which you were randomly allocated at the start of today's proceedings. I would also like to stress that your payment does not depend upon your questionnaire answers. When you are done with the questionnaire please save the document.

[Untimed (allow around 10 minutes): Questionnaire]

I will ask you to remain seated since you will need to sign a receipt for your payments. Many thanks for taking part in today’s session.

**PANAS Questionnaire**

This scale consists of a number of words that describe different feelings and emotions. Read each item and then list the number from the scale below next to each word. Indicate to what extent you feel this way right now, that is, at the present moment.

Scare: 1 - Very Slightly, 2- A little, 3- Moderately, 4 -Quite a bit 5- Extremely

1. Interested: 1 2 3 4 5

2. Distressed: 1 2 3 4 5

3. Excited: 1 2 3 4 5

4. Upset: 1 2 3 4 5

5. Strong: 1 2 3 4 5

6. Guilty: 1 2 3 4 5

7. Scared: 1 2 3 4 5

8. Hostile: 1 2 3 4 5

9. Enthusiastic: 1 2 3 4 5

10. Proud: 1 2 3 4 5

11. Irritable: 1 2 3 4 5

12. Alert: 1 2 3 4 5

13. Ashamed: 1 2 3 4 5

14. Inspired: 1 2 3 4 5

15. Nervous: 1 2 3 4 5

16. Determined: 1 2 3 4 5

17. Attentive: 1 2 3 4 5

18. Jittery: 1 2 3 4 5

19. Active: 1 2 3 4 5

20. Afraid: 1 2 3 4 5

**Socio-Economic Status Questionnaire**

1. Gender: M F

2. Age:

3. Actual Education Degree: Bachelor Master

4. Do you come from a rural area? Y N

5. How do you finance your studies? Scholarship , Loan , Parents , Side Job, Other.

6. In which country where you born?

7. Have you ever lived in a developing country? Y N

8. How long have you lived in a developing country (years)?

9. Have you traveled to a developing country? Y N

10. How many times have you traveled to a developing country?

11. Have you ever worked for a charity organization that helps poor people? Y N

12. How many real estate properties do your family own?

13. What is the highest degree that your father achieved? Elementary School, High School, University, Master, PhD.

14. What is the highest degree that your mother achieved Elementary School, High School, University, Master, PhD.

15. How many cars do your parents own?

16. What is the current labor status of your father: Unemployed, Self-Employed, Partime, Employed, Fulltime Employed, Retired.

17. What is the current labor status of your mother: Unemployed, Self-Employed, Partime, Employed, Fulltime Employed, Retired.
